# Supplementary figures and images for: All-atom simulation of the HET-s prion replication
Source: PLoS Comput Biol. 2020 Sep 18;16(9):e1007922. doi: 10.1371/journal.pcbi.1007922 (PMC7526898; doi:10.1371/journal.pcbi.1007922)

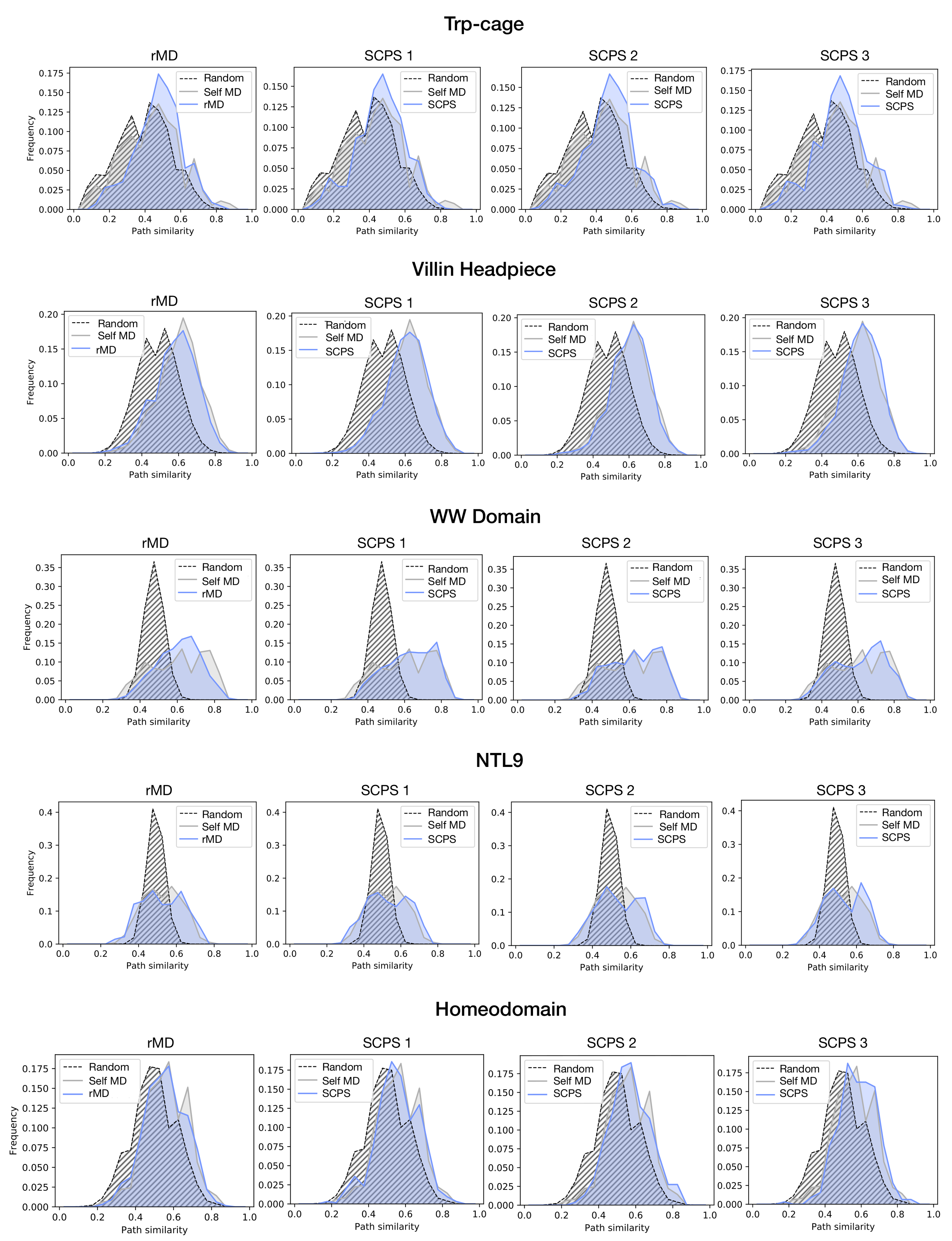

Supplement: S1 Fig — In each graph, three path similarity distributions are represented: (i) the path similarity distribution obtained by comparing the order of native contact formation between biased folding trajectories (rMD or SCPS) with the plain-MD folding trajectory (defined as cross-similarity Ri, blue; where i is the iteration number); (ii) the path similarity distribution computed by comparing the order of native contact formation between the folding plain-MD trajectories within themselves (defined as self-similarity, A, grey); (iii) the path similarity obtained by comparing the plain-MD trajectories with random sequences of native contact formation (defined as random, Rr, dashed line). (TIF) [file pcbi.1007922.s001.tif]

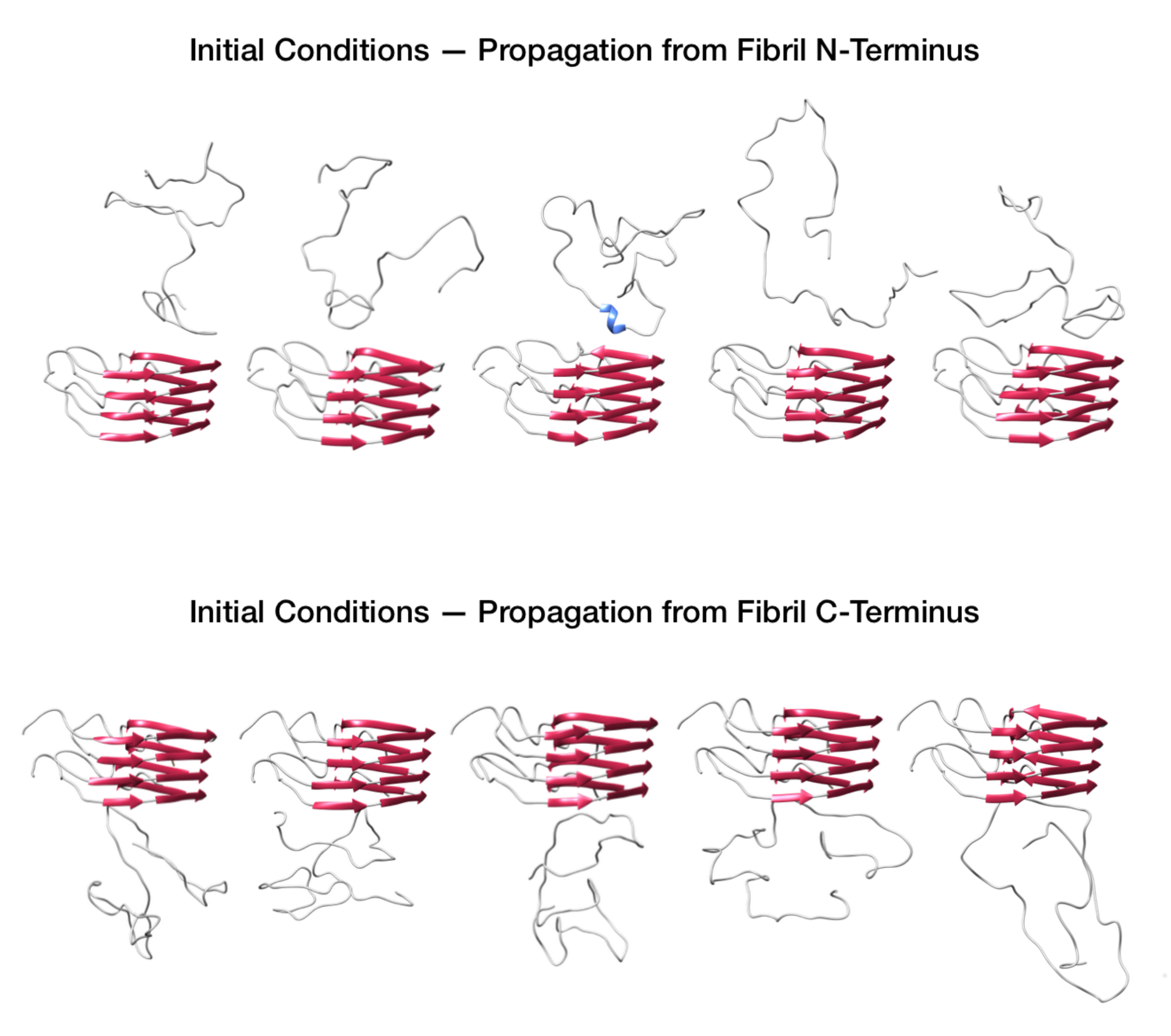

Supplement: S2 Fig — The initial conditions used to generate the propagation pathways are reported. Initial conditions for simulating propagation from the fibril N-terminus were obtained by performing high-temperature MD, introducing positional restraints on heavy atoms on the two C-terminal monomers. Initial conditions for simulating propagation from the fibril C-terminus were obtained by performing high-temperature MD, introducing positional restraints on heavy atoms on the two N-terminal monomers. (TIF) [file pcbi.1007922.s002.tif]

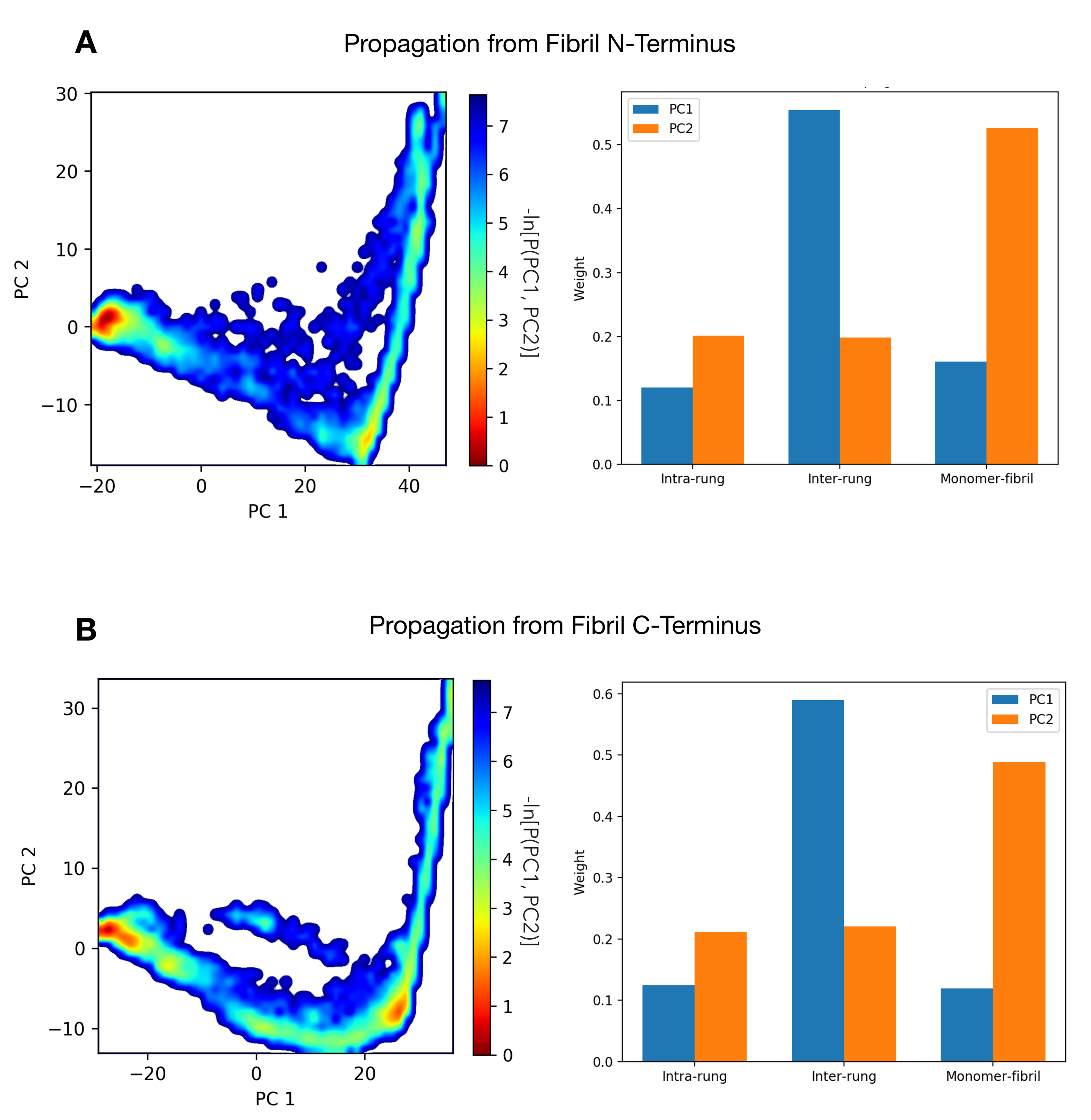

Supplement: S3 Fig — Graphs on the left represent the free energy landscape in the principal component plane of the trajectories propagating from the fibril N-terminus (A) and C-terminus (B), respectively. Bar plots on the right show the contribution of the contact-type sets for the two principal components. (TIF) [file pcbi.1007922.s003.tif]

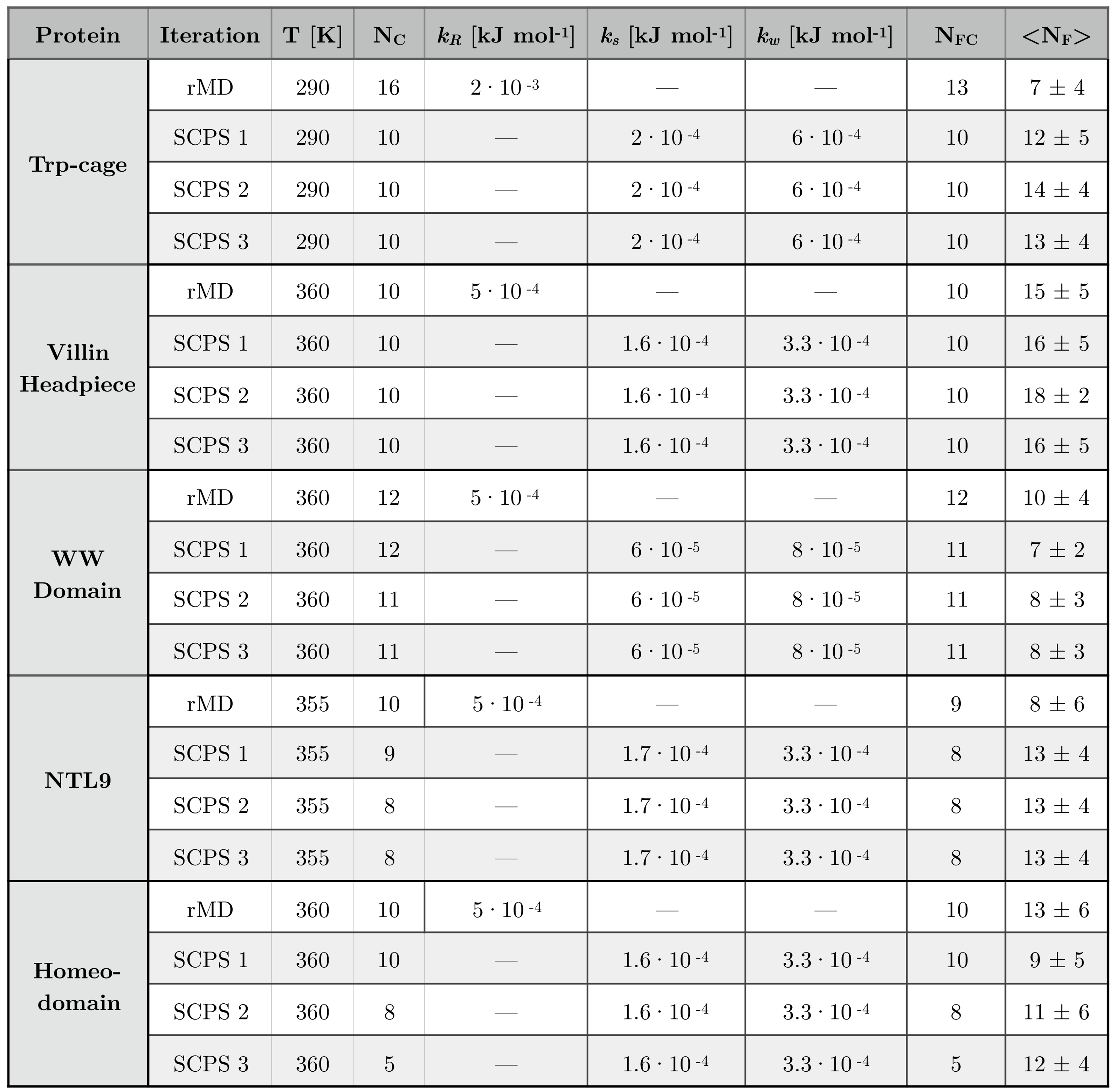

Supplement: S1 Table — In this table, additional information regarding the folding simulations are reported. NC is the number of initial conditions, T is the simulations temperature, kR is the ratchet force constant, ks and kw are the SCPS force constants, NFC is the number of sets (each set start from a different initial condition) for which at least one folding event is observed and < NF > is the average number of folding trajectories for each set. (TIF) [file pcbi.1007922.s004.tif]
