# Supplementary material for: All-atom simulation of the HET-s prion replication
Source: PLoS Comput Biol. 2020 Sep 18;16(9):e1007922. doi: 10.1371/journal.pcbi.1007922 (PMC7526898; doi:10.1371/journal.pcbi.1007922)
Supplement: Attachment — Submitted filename: Reply to referee.pdf [file pcbi.1007922.s008.pdf]

REPLY TO THE REFEREE REPORT

We would like to thank the referee for his/ her competent and careful review. We feel that the constructive criticisms raised in the report have helped us improve the manuscript

COMMENT:

Although both messages are potentially compelling, I felt it was unclear which one was intended to be the main emphasis in the current version of the paper. For this work to succeed as a methods paper (message 1), it would benefit from more technical detail (e.g. how SCPS works, which at the moment is not fully clear to a reader who hasn't read previous papers), discussion of general considerations related to simulating amyloid systems with this method, and potentially some sort of cross validation to convince readers that this method, as applied to amyloid fibers, yields sensible predictions. On the other hand, I felt the paper as it stands lacks sufficient discussion of specific, empirically-verifiable predictions that is necessary to make this work a complete biophysical picture (message 2).

REPLY:

We agree with the referee. In principle, focusing on phenomenology may broaden the potential readership of this paper. However, we think that the most relevant result of this work is to show that the SCPS method provides an efficient theoretical/computational platform to investigate a wide range of prion propagation processes and, more generally, amyloidogenic misfolding reactions. To date, the only application in literature of the SCPS protocol was a proof-of-principle study, that was performed on a mini-protein using an implicit solvent model. For this reason, we decided to focus this work on validating SCPS method and discussing its applicability to study misfolding events. In our future papers, we will focus on phenomenology and will be in a condition to rely on this publication for all technical details.

COMMENT:

Fig. 3: I found it interesting that, whereas the C terminal hairpin has significant variation in its order of secondary structure formation (large error bars), the N-terminal hairpin appears to form secondary structure in a much more stereotypical fashion. Namely residues 238-241 usually seem to form prior to 226-234, regardless of the direction of propagation. Why may this may be so? Would you expect that mutating these former residues may disrupt proper fibril formation/propagation?

REPLY

We thank the reviewer for this very interesting observation. We do not have an explanation for such behavior, but it certainly raises an hypothesis potentially testable with mutagenesis experiments. We have now inserted a comment regarding this point into the text (lines 543-548 of the revised version).

COMMENT:

The authors mention at the end of the results section (subsection "All-atom reconstruction…") that the results are consistent with experimental findings insofar as they support a model in which fibril formation in which the presence of a seed significantly speeds up amyloid growth by eliminating the nucleation barrier. Although this is certainly a reasonable mechanism, it is unclear to me that these results actually show this, since as far as I understand, this method cannot shed light on kinetics and can only provide a reasonable C.V. In order to address kinetics, wouldn't it be necessary to compute the equilibrium folding landscape along this C.V. to get the barrier height (e.g. using umbrella biasing)? Moreover, at the moment, all simulations are done in the presence of an existing fibril, so I'm not sure how a comparison with seed-free conditions can be made.

REPLY:

The referee is indeed right when stating that the SCPS cannot be used to directly estimate free-energy barriers, thus this method cannot yield direct predictions about kinetics. The comparison with experiments is indeed far more indirect and qualitative. Namely, the statistical models introduced in Ref. [21]and [25] of the revised version predict that, in the presence of a fibril seed, the dominant reaction pathway is one in which the prion rungs misfold one-by-one using the rungs in the fibril as template. This theoretical prediction is clearly observed in our SCPS simulation. The stochastic model of Ref. [26] also implies that misfolding should be highly promoted by the presence of a seed fibril. This prediction is verified experimentally. We have illustrated this point more clearly in lines 713–720 of the revised version of our manuscript.

COMMENT:

Although this work proposes a mechanism for growth via either end of the fibril (N or C terminus), it is not obvious to me that both directions will be equally favorable under unbiased (equilibrium) conditions. Is it possible that, under experimental conditions or in vivo, one of the two directions will have a significantly higher free energy barrier than the other, and thus be disfavored?

REPLY:

This is another valid point raised by the reviewer. While our method cannot distinguish a precise directionality of the growth (N or C terminus), it is possible that additional variables occurring in vivo may determine it. For example, the presence of specific factors capping one of the two ends would exert such an effect. We elaborate this important point in lines 732-739 of the revised manuscript

ADDITIONAL COMMENTS:

1. *In Figs. 2 and 4, unless I misunderstood something, I found that plotting these "landscapes" in units of kT to be misleading since, as the authors themselves comment in the methods section, these simulations are not at equilibrium. It is not clear to me in what*

sense these values can be interpreted as free energies. I would simply express refer to them as (negative) log probabilities.

We have implemented this change in the figure captions.

2. *In Fig. 3, is it correct to interpret the sequence of secondary structure formation as the sequence with which monomer residues dock to the fibril? I would imagine so since forming beta secondary structure should be entropically unfavorable in undocked state.*

   Yes. We clarified this point in lines 171-172

3. *Methods section: Please define Z_m and theta as the Heaviside function immediately after equation 1. Likewise immediately after equation 2, please indicate that i and j index atoms.*

   We have implemented these changes

4. *Methods section: The authors mention that lamda is set as the inverse of the average distance between these contact maps. But as I understand from previous work, S and W work best as order parameters in the limit where lambda is large. So can the authors comment on whether this inverse average distance is indeed a large value?*

   The referee is right about this point. In principle, the collective variable $s_\lambda$represents a progress variable along the mean-path only in the large lambda limit. However, in numerical simulation the choice of this parameter must be done in order to ensure computational efficiency and numerical reliability. The choice reported in the paper enables us to fulfill both requirements. We have added a paragraph into the text to clarify this point (lines 830-831).

5. *A few grammar mistakes should be corrected. Possible corrections shown in red.*
   a. *Abstract: Indeed, in such an approach the applied biasing force is not heuristically postulated but rather is calculated through an iterative refinement procedure.*

      We have re-written part of the abstract to avoid providing too much detail about the differences between different enhanced sampling methods.

   b. *Introduction: Thus, SCPS provides a much better tool than BF to investigate structural reactions in which the reaction coordinate is poorly known, including prion propagation.*

      We have corrected this.

   c. *Results: In Fig 3, we show the median value of the reaction progress variable Q at which each residue assumes the β-strand conformation.*

We have corrected this.

d. *Methods: In turn, an updated version of the s.(X), and w.(X) collective variables is computed.*

We have corrected this.

e. *Fig. 3 legend panel: The sentence "On the contrary, in the propagation starting from the fibril C-terminus" is incomplete. Perhaps change to "The opposite sequence of events is observed when propagation starts from the fibril C-terminus".*

We have followed this suggestion.
