# Supplementary material for: All-atom simulation of the HET-s prion replication
Source: PLoS Comput Biol. 2020 Sep 18;16(9):e1007922. doi: 10.1371/journal.pcbi.1007922 (PMC7526898; doi:10.1371/journal.pcbi.1007922)
Supplement: Attachment — Submitted filename: AB_review.docx [file pcbi.1007922.s007.docx]

In this work, Terruzzi et. al. make use of Self-Consistent Path Sampling (SCPS) Molecular Dynamics simulations to model the amyloid propagation mechanism for the prion domain of the fungal protein Het-s. They paper presents two main messages, namely:

1. The SPCS methodology can be deployed to simulate complex amyloid systems in a computationally-efficient manner without requiring *a priori* knowledge of the optimal collective variable (which is important in other methods such as rMD).
2. This method generates a novel biophysical picture of how Het-s amyloid formation is propagated, whereby Het-s monomers can dock onto either terminus of the growing Het-s fibril—this is contrast to the homologous fungal PrP prion where amyloid elongation is unidirectional. Upon docking of the first beta rung of the monomer to the growing fibril, the second monomer rung proceeds to dock via chain reorganization.

Although both messages are potentially compelling, I felt it was unclear which one was intended to be the main emphasis in the current version of the paper. For this work to succeed as a methods paper (message 1), it would benefit from more technical detail (e.g. how SCPS works, which at the moment is not fully clear to a reader who hasn’t read previous papers), discussion of general considerations related to simulating amyloid systems with this method, and potentially some sort of cross validation to convince readers that this method, as applied to amyloid fibers, yields sensible predictions. On the other hand, I felt the paper as it stands lacks sufficient discussion of specific, empirically-verifiable predictions that is necessary to make this work a complete biophysical picture (message 2).

While I could envision either strategy working, my subjective sense is that message 2 would appeal to a broader audience, in which case the authors may consider moving some details regarding computational methods to the methods/Supplementary sections, such that they are accessible to an interested computational reader, but do not distract from the main points that an experimental reader would be most interested in. In addition, the emphasis on experimentally-verifiable predictions should be increased. A few possible examples include:

1. Fig. 3: I found it interesting that, whereas the C terminal hairpin has significant variation in its order of secondary structure formation (large error bars), the N-terminal hairpin appears to form secondary structure in a much more stereotypical fashion. Namely residues 238-241 usually seem to form prior to 226-234, regardless of the direction of propagation. Why may this may be so? Would you expect that mutating these former residues may disrupt proper fibril formation/propagation?
2. The authors mention at the end of the results section (subsection “All-atom reconstruction…”) that the results are consistent with experimental findings insofar as they support a model in which fibril formation in which the presence of a seed significantly speeds up amyloid growth by eliminating the nucleation barrier. Although this is certainly a reasonable mechanism, it is unclear to me that these results actually show this, since as far as I understand, this method cannot shed light on kinetics and can only provide a reasonable C.V. In order to address kinetics, wouldn’t it be necessary to compute the equilibrium folding landscape along this C.V. to get the barrier height (e.g. using umbrella biasing)? Moreover, at the moment, all simulations are done in the presence of an existing fibril, so I’m not sure how a comparison with seed-free conditions can be made.
3. Although this work proposes a mechanism for growth via either end of the fibril (N or C terminus), it is not obvious to me that both directions will be equally favorable under unbiased (equilibrium) conditions. Is it possible that, under experimental conditions or *in vivo*, one of the two directions will have a significantly higher free energy barrier than the other, and thus be disfavored?

Additional comments:

1. In Figs. 2 and 4, unless I misunderstood something, I found that plotting these “landscapes” in units of kT to be misleading since, as the authors themselves comment in the methods section, these simulations are not at equilibrium. It is not clear to me in what sense these values can be interpreted as free energies. I would simply express refer to them as (negative) log probabilities.
2. In Fig. 3, is it correct to interpret the sequence of secondary structure formation as the sequence with which monomer residues dock to the fibril? I would imagine so since forming beta secondary structure should be entropically unfavorable in undocked state.
3. Methods section: Please define Z_m and theta as the Heaviside function immediately after equation 1. Likewise immediately after equation 2, please indicate that i and j index atoms.
4. Methods section: The authors mention that lamda is set as the inverse of the average distance between these contact maps. But as I understand from previous work, S and W work best as order parameters in the limit where lambda is large. So can the authors comment on whether this inverse average distance is indeed a large value?
5. A few grammar mistakes should be corrected. Possible corrections shown in red.
   a. **Abstract**: Indeed, in such an approach, ~~the CV~~ ~~where~~ the applied biasing force

~~is applied~~ is not heuristically postulated but rather is calculated through an iterative refinement procedure.

b. **Introduction:** Thus, SCPS provides a much better tool than BF to investigate structural reactions in which the reaction coordinate is poorly known, ~~thus~~ including prion propagation.
c. **Results:** In Fig 3, we show the median value of the reaction progress variable Q at which each residue assumes the β-strand conformation.
d. **Methods**: In turn, an updated version of the s.(X), and w.(X) collective variables is computed.
e. **Fig. 3 legend panel:** The sentence “On the contrary, in the propagation starting from the fibril C-terminus” is incomplete. Perhaps change to “The opposite sequence of events is observed when propagation starts from the fibril C-terminus”.
